# Reductive Treatment of Pt Supported on Ti_0.8_Sn_0.2_O_2_-C Composite: A Route for Modulating the Sn–Pt Interactions

**DOI:** 10.3390/nano13152245

**Published:** 2023-08-03

**Authors:** Cristina Silva, Khirdakhanim Salmanzade, Irina Borbáth, Erzsébet Dódony, Dániel Olasz, György Sáfrán, Andrei Kuncser, Erzsébet Pászti-Gere, András Tompos, Zoltán Pászti

**Affiliations:** 1 Institute of Materials and Environmental Chemistry, Research Centre for Natural Sciences, Magyar Tudósok Körútja 2, H-1117 Budapest, Hungary; flakcriss45@gmail.com (C.S.); ksalmanzade5@gmail.com (K.S.); borbath.irina@ttk.hu (I.B.); tompos.andras@ttk.hu (A.T.); 2 Department of Physical Chemistry and Materials Science, Faculty of Chemical Technology and Biotechnology, Budapest University of Technology and Economics, Műegyetem rkp. 3., H-1111 Budapest, Hungary; 3 Institute for Technical Physics and Materials Science, Centre for Energy Research, Konkoly-Thege Miklós út 29-33, H-1121 Budapest, Hungary; dodonye@gmail.com (E.D.); olasz.dani96@gmail.com (D.O.); safran.gyorgy@ek-cer.hu (G.S.); 4 National Institute of Materials Physics, 405A Atomistilor Street, 077125 Magurele, Romania; andrei.kuncser@infim.ro; 5 Department of Pharmacology and Toxicology, University of Veterinary Medicine, István utca 2, H-1078 Budapest, Hungary; gere.erzsebet@univet.hu

**Keywords:** electrocatalyst, mixed oxide–carbon composite, platinum–tin interaction, strong metal-support interaction

## Abstract

The composites of transition metal-doped titania and carbon have emerged as promising supports for Pt electrocatalysts in PEM fuel cells. In these multifunctional supports, the oxide component stabilizes the Pt particles, while the dopant provides a co-catalytic function. Among other elements, Sn is a valuable additive. Stong metal-support interaction (SMSI), i.e., the migration of a partially reduced oxide species from the support to the surface of Pt during reductive treatment is a general feature of TiO_2_-supported Pt catalysts. In order to explore the influence of SMSI on the stability and performance of Pt/Ti_0.8_Sn_0.2_O_2_-C catalysts, the structural and catalytic properties of the as prepared samples measured using XRD, TEM, XPS and electrochemical investigations were compared to those obtained from catalysts reduced in hydrogen at elevated temperatures. According to the observations, the uniform oxide coverage of the carbon backbone facilitated the formation of Pt–oxide–C triple junctions at a high density. The electrocatalytic behavior of the as prepared catalysts was determined by the atomic closeness of Sn to Pt, while even a low temperature reductive treatment resulted in Sn–Pt alloying. The segregation of tin oxide on the surface of the alloy particles, a characteristic material transport process in Sn–Pt alloys after oxygen exposure, contributed to a better stability of the reduced catalysts.

## 1. Introduction

Due to their specific properties, polymer electrolyte membrane fuel cells (PEMFCs) comprise the most important type of fuel cells for small or medium-scale stationary, mobile or portable hydrogen-based electricity generation [1]. The still unsurpassed properties of platinum supported on high surface area carbon (Pt/C) make it the most widely used electrocatalyst both at the anode and cathode side of PEMFCs. However, due to its scarce availability, Pt is an expensive and critical raw material. At the same time, the corrosion of the Pt/C catalysts results in a continuous performance decrease [2]. Thus, catalyst degradation is the limiting factor determining the lifetime of the cell. This issue, along with the limited performance of the Pt/C catalyst in the oxygen reduction reaction (cathode process), is typically compensated by high Pt loads, especially at the cathode electrode. Thus, the electrocatalysts are responsible for approx. 30–40% of the price of the PEMFC [3]. Understandably, significant efforts are focused on either replacing Pt with cheaper alternatives or enhancing the activity and longevity of the Pt catalyst, which could decrease the Pt load and, in turn, the price of the electrodes [4].

A possibility for modulating the properties of the Pt catalyst is the dilution of the active metal with an appropriate co-catalytic component in an alloy-type bimetallic system [5,6,7]. In such catalysts, the activity and selectivity are determined not only by the chemical nature of the alloying element. Instead, structural factors, such as the distribution of the dopant (e.g., randomly alloyed, core-shell arrangement, ordered intermetallic compound) or the morphology of the catalyst (determined by both the nature of the metal–metal interaction and the synthesis method), are also very important [8,9]. Among other alloying elements, tin was identified as a particularly versatile material [10,11]. The Sn–Pt system was found to be useful both at the anode (providing tolerance against CO poisoning in direct methanol cells or if hydrogen obtained from reforming reactions is used) and the cathode (providing good activity in the oxygen reduction reaction) of PEMFCs. The co-catalytic effect of Sn is generally attributed to the easy formation of reactive OH species on Sn sites, which facilitate the oxidation of poisoning CO intermediates adsorbed on Pt according to the well-known bifunctional mechanism [12,13]. It was also suggested that SnO_x_ species that form on the surface of the alloy nanoparticles under reaction conditions can protect Pt sites from oxidation and subsequent dissolution [9]. In particular, the Pt_3_Sn ordered intermetallic compound phase appeared to be the most active and stable catalyst [14,15,16,17]. Nevertheless, the synthesis of the supported Sn–Pt catalysts with tin exclusively connected to Pt is not straightforward [18], although special methodologies are available for the preparation of Pt_3_Sn on carbon or oxides [19].

Another strategy for enhancing the longevity of the PEMFC electrocatalysts is to replace the corrosion-sensitive carbon support with a more stable oxide–carbon composite material. The basic idea is to combine the nanoparticle stabilizing effect of inorganic oxides with the good conductivity and high specific surface area of traditional or novel carbon materials. As emphasized by numerous examples in the literature, the strong interaction between the active metal and the composite support arising at metal–oxide–carbon triple junctions is a key factor in enhancing the stability- and/or activity-related properties of the system [20,21].

TiO_2_, which is widely used in heterogeneous catalysis and have an inherently higher stability than carbon in acidic and oxidizing environments, is a good candidate for this purpose. As TiO_2_ can also incorporate dopants such as W [22], Mo [23], Nb [24] or Sn [25], the co-catalytic properties of these oxophilic elements can also be exploited for electrocatalytic purposes. Indeed, Pt electrocatalysts supported on W- or Mo-doped rutile–carbon composites turned out to be highly CO-tolerant while showing an improved long-term stability compared to Pt/C references not only in three-electrode electrochemical tests [26,27] but also in fuel cell tests [28,29]. It should be noted that characteristic performance differences were identified between the catalysts with different mixed oxide/carbon ratios [27], and an optimal ratio should be determined depending on the purpose of the study. Surface chemical investigations suggested that active sites responsible for the CO-tolerant behavior were formed around the perimeter of the Pt particles at the oxide–carbon–Pt triple junctions [30]. Nevertheless, the leaching of metal ions from the catalyst under the working conditions of the cell is always a concern as these species can interfere with the proton conducting property of the membrane [31]. Our previous results indicated that co-catalyst metal dissolution can be prevented by incorporating the dopant into the substitution sites of the TiO_2_ matrix, which also increases the conductivity of the oxide.

Studies using Pt/SnO_2_ catalysts identified the stabilizing effect of the oxide without a loss in the catalytic performance compared to Pt/C [32], which can be attributed to a strong interaction between the Pt particles and the oxide [33]. These interactions can range from modulating the shape and structure of the Pt particle, often resulting in the formation of Pt clusters with strained lattices, to the complete encapsulation of the Pt element by an ultrathin layer of the partially reduced oxide, similar to the classical strong metal-support interaction (SMSI) phenomenon [34].

In fact, SMSI-like effects are often encountered in thermal catalysis at elevated temperatures between reducible metal oxides and platinum group metal nanoparticles. Their influence on the properties of the catalyst, especially on its selectivity, is well documented [35,36,37]. In the electrocatalysis field, the SMSI-related phenomena become significant due to their potential for enhancing the catalytic performance [36,38] and improving the stability [39,40,41,42]. Importantly, the spontaneous development of such phenomena at the typically low temperatures of electrochemical processes is improbable, as the required material transport processes are thermodynamically favored but kinetically hindered. Accordingly, in electrocatalysis, evoking the SMSI state can be achieved as a specific step of the catalyst preparation route via thermal pre-treatment (typically in reducing [32,34] but in some systems in oxidizing [38] atmospheres) or the deposition of specific adsorbates to direct the encapsulation [43]. Despite the promising benefits of SMSIs in electrocatalysis, its application has been limited due to the challenges associated with creating a well-regulated interaction between the materials. The controlling factors, such as the electron transfer, mass transfer and the thickness of the encapsulation layer, are crucial for achieving uniformity in the electrocatalytic surface [43,44].

In the case of co-catalyst-doped Ti_(1−x)_M_x_O_2_-C (M = W, Mo, Sn, Nb) composite-supported catalysts, Pt and the oxide component are liable for a strong metal-support interaction. In a previous study, the stability enhancement of a Pt/Ti_0.8_Mo_0.2_O_2_-C catalyst after reductive pre-treatment was attributed to the migration of (ionic) Mo species to the surface of the Pt particles [45]. Similarly, the reductive treatment of Pt/SnO_2_ catalysts was identified as a promising method for improving their stability [46] or modulating their surface properties [47]. However, the nature of the Pt-support interactions in Sn-doped rutile–carbon composite-supported electrocatalysts, involving material transport processes and structure formation via reductive pre-treatment, is still an open question, one that is investigated in the present contribution. A comparison of the structural, physicochemical and electrochemical properties of the as prepared and reduced Pt/Ti_0.8_Sn_0.2_O_2_-C catalysts was conducted in order to assess the potential of reductive treatment as a strategy for improving the electrocatalytic performance of these tin-containing composite-supported systems.

## 2. Materials and Methods

### 2.1. Materials

The Ti_0.8_Sn_0.2_O_2_-C composite supports were prepared using Black Pearls 2000 active carbon (Cabot Corporation, Boston, MA, USA). For the composite synthesis, titanium isopropoxide (Sigma-Aldrich, St. Louis, MO, USA, 97%), tin (IV) chloride-5-hydrate (Honeywell Riedel-de Haën GmbH, Seelze, Niedersachsen Germany, 98%), nitric acid (65%, a.r., Molar Chemicals, Halásztelek, Hungary) and ultrapure water (18 MΩ cm, produced by Millipore equipment, (Burlington, MA, USA)) were used. The Pt precursor for electrocatalyst synthesis was H_2_PtCl_6_·6H_2_O (Sigma-Aldrich, St. Louis, MO, USA, 37.5% Pt). Further chemicals used for the Pt loading were ethanol (99.55%), ethylene glycol (99.8%), HCl (37%) and NaBH_4_ (99.95%) (all obtained from Molar Chemicals). The catalyst ink for the electrochemical studies was prepared using a Nafion solution (DuPont™ Nafion^®^ PFSA Polymer Dispersions DE 520, The Chemours Company, Wilmington, DE, USA), isopropanol (Molar Chemicals) and ultrapure water (see above).

### 2.2. Preparation of the Pt/Ti_0.8_Sn_0.2_O_2_-C Composite-Supported Electrocatalysts

**Scheme 1:**
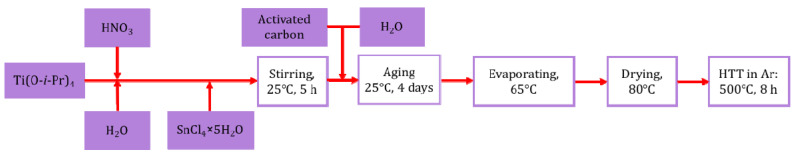
Synthesis of the Ti_0.8_Sn_0.2_O_2_-C composite support.

The Ti_0.8_Sn_0.2_O_2_-C composite support containing 75 wt.% oxide and 25 wt.% Black Pearls 2000 carbon was prepared using a variant of the multistep sol–gel synthesis method that was developed for the production of the Ti_0.8_Mo_0.2_O_2_-C composites [27]. The key steps of the process are shown in Scheme 1 [48].

The first step was the preparation of a transparent Ti sol by adding 1.889 mL of titanium isopropoxide to an acidic aqueous solution, followed by the introduction of the Sn precursor tin (IV) chloride pentahydrate (559.3 mg). After stirring the mixture at room temperature, 250 mg of active carbon in water was added and stirring was continued for 4 days to deposit the rutile nuclei on the carbon backbone. Then, the solvent was evaporated and the material was washed and dried at 80 °C. Finally, a high-temperature treatment step (HTT, in argon flow at 500 °C for 8 h) was applied to improve the crystallinity of the oxide.

**Scheme 2:**
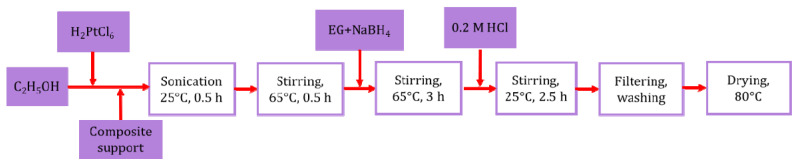
Pt loading of the Ti_0.8_Sn_0.2_O_2_-C composite.

Pt (20 wt.%) was introduced using a modified NaBH_4_-assisted ethylene glycol (EG) reduction–precipitation technique, as shown in Scheme 2 [49].

An amount of 0.643 mmol H_2_PtCl_6_·6H_2_O was dissolved in 50 mL of ethanol, and a 200 mg composite support was added to the solution. A solution containing 7.8 mmol NaBH_4_ with 3.7 mL ethylene glycol was dropwise added at 65 °C during continuous stirring. After 3 h of stirring, 15 mL of 0.2 M HCl was added to the suspension, which was then agitated for a further 2.5 h at room temperature to deposit the platinum particles onto the support material. The Pt loading was completed by washing the catalyst three times, and then the material was filtered, centrifuged and dried at 80 °C overnight.

### 2.3. Reductive Pre-Treatment

After the catalyst preparation, a fraction of the material was annealed in a hydrogen flow at 200 °C for 2 h in order to induce the metal-support interactions. This temperature was determined as a compromise between the extent of the surface chemistry changes and the growth of platinum particles, and was also guided by our previous experience with supported Sn–Pt alloy catalysts [19].

### 2.4. Phase Composition and Morphology

The synthesis of the composite was followed, and the catalysts in the as prepared and reduced state were characterized via X-ray diffraction using a Philips model X’PERT MPD and PW 3710-based PW 1050 Bragg–Brentano parafocusing goniometer (Philips Analytical, Almelo, The Netherlands) with CuKα radiation, a graphite monochromator and a proportional counter. The microstructure was investigated via transmission electron microscopy (TEM) using a FEI Titan Themis 200 kV C_s_—corrected TEM with 0.09 nm HRTEM resolution (ThermoFisher Scientific, Waltham, MA, USA). The scanning transmission electron microscopy (STEM) capabilities of the microscope were employed for recording high-resolution elemental maps using the energy dispersive spectroscopy (EDS) technique. The Pt particle distribution was determined by measuring the diameters of at least 800 randomly selected metal particles in five micrographs of each sample taken from non-aggregated areas. The evaluation and processing of the high-resolution micrographs was performed using the ImageJ software [50].

### 2.5. Surface Chemical Characterization

The surface composition of the as prepared and reduced catalysts as well as the chemical states of their components were investigated via X-ray photoelectron spectroscopy (XPS) using an Omicron EA 125 electron spectrometer (Omicron Nanotechnology GmbH, Taunusstein, Germany) operated in the constant analyzer energy mode (pass energy 30 eV, resolution around 1 eV). The photoelectrons were excited using MgKα radiation. In order to follow the effect of the reductive pre-treatment on the surface chemistry of the as prepared catalyst, a hydrogen exposure series in 100 mbar H_2_ for 1 h at different temperatures (room temperature, 100 °C, 200 °C and 300 °C) was performed in the high-pressure/high-temperature treatment chamber of the spectrometer without exposure to ambient conditions. This treatment was completed by re-oxidation in O_2_ or air at room temperature. Spectral information was collected after all the treatment steps. The spectra were processed using the software Casa XPS [51] by fitting the data to a combination of Gaussian–Lorentzian peaks with linear or Shirley backgrounds. The quantitative analysis was performed using the software XPS MultiQuant [52,53], assuming there was a homogeneous distribution of the components as described in our previous studies.

The surface sensitivity of the XPS method is determined by the inelastic mean free path of the photoelectrons, which is dependent on the kinetic energy of the electrons. In general, the mean free path curve has a broad minimum approaching 0.4–0.5 nm, i.e., one or two monolayers around 40–100 eV then it gradually increases, reaching 2–3 nm around 1000 eV [54]. The major contribution to the intensity of a given photoelectron peak arises from the atoms located no deeper than one to two times the inelastic mean free path. Qualitatively, it means that if a surface is covered by a thin overlayer, the intensity of the lower kinetic energy peaks is suppressed to a much higher extent than that of the higher kinetic energy peaks in comparison to the case of the uncovered surface. In this sense, the intensity ratio of a low and high kinetic energy Pt peak can serve as an indicator of the buried or exposed state of the surface Pt species. In order to exploit this effect for assessing the state of the Pt particles, the spectra of a low energy Pt feature (Pt NOO Auger peak at 64 eV kinetic energy, mean free path around 0.4 nm) were recorded simultaneously with the Pt 4f photoelectron peak (at 1180 eV kinetic energy for MgKα excitation, mean free path around 3 nm) in the same mode of the energy analyzer (fixed retard ratio set to 7) and the intensities were determined as the area under the peak after background subtraction.

### 2.6. Electrochemical Characterization

The as prepared and reduced electrocatalysts were investigated using cyclic voltammetry (CV) and CO_ad_ stripping voltammetry that were completed by a short (500 CV cycles) and long (5000 CV cycles) stability assessment. The measurements were performed in a conventional three-electrode electrochemical glass cell using a Biologic SP150 potentiostat and the EC-LAB software package (version 11.31) (BioLogic, Seyssinet-Pariset, France). The working electrode (glassy carbon GC d = 0.3 cm, geometric surface A = 0.0707 cm^2^) was polished before each test to remove any traces of organic impurities. The catalyst ink was prepared using the standard method described in our previous work [27]. The applied electrolyte was 0.5 M H_2_SO_4_. Pt was used as counter electrode and the reference electrode was a reversible hydrogen electrode (RHE). All the potentials were given on RHE scale.

The electrochemically active surface area (ECSA) of the electrocatalysts was determined from the charge needed for oxidation under potentially deposited hydrogen [55] using a conventional baseline correction as described in our previous studies [26,27].

## 3. Results and Discussion

First, a structural description of the as prepared and reduced Pt/Ti_0.8_Sn_0.2_O_2_-C electrocatalysts will be given. Afterwards, the surface chemistry peculiarities of the catalysts will be explored, and finally their electrochemical properties will be discussed and related to the presented structural and surface chemical information.

### 3.1. Structural Characteristics of the as Prepared and Reduced Pt/Ti_0.8_Sn_0.2_O_2_-C Catalysts

**Figure 1:**
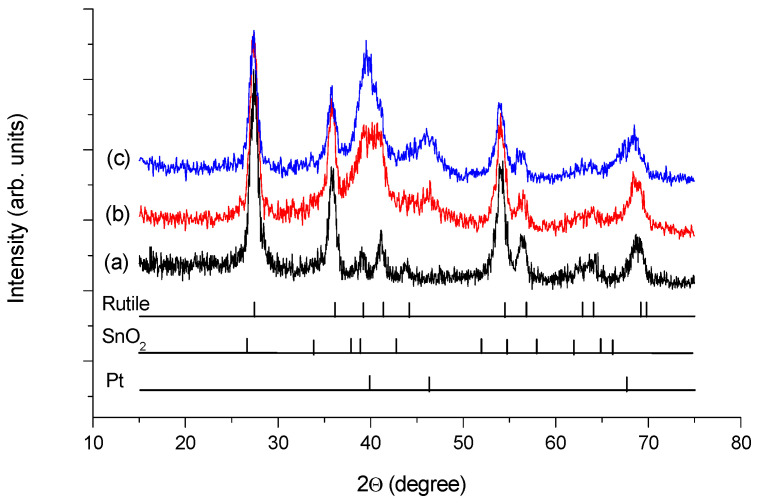
X-ray diffraction patterns of (**a**) the Ti_0.8_Sn_0.2_O_2_-C composite support, (**b**) the as prepared Pt/Ti_0.8_Sn_0.2_O_2_-C electrocatalyst and (**c**) the Pt/Ti_0.8_Sn_0.2_O_2_-C electrocatalyst reduced at 200 °C in a hydrogen flow. The expected reflection positions of rutile (JCPDS 21-1276), SnO_2_ (JCPDS 41-1445) and platinum (JCPDS 04-0802) are shown as bar graphs.

The composition and structure of the samples were investigated using XRD and TEM/STEM that were completed using elemental mapping. The X-ray diffraction patterns of the bare Ti_0.8_Sn_0.2_O_2_-C composite support, the as prepared electrocatalyst and its reduced counterpart are shown in Figure 1.

The diffraction patterns demonstrated the exclusive presence of a rutile-type oxide. No indication of another phase-like crystalline SnO_2_ or anatase-type TiO_2_ was observed. The lattice parameters obtained from the reflection angles are listed in Table 1. A slight increase with respect to the unit cell dimensions of pure rutile TiO_2_ was caused by the incorporation of Sn into the lattice.

The extent of the tin incorporation estimated from the lattice parameter changes using Vegard’s law [56,57] is also given in Table 1. The data indicated an approx. 10–15% doping level. As the estimated extent of the Sn incorporation was lower than the nominal tin content, the formation of a disordered tin-oxide containing phase seemed to be probable. According to the Scherrer formula, the oxide crystallite sizes were around 10 nm.

Pt loading caused no obvious change in the rutile reflections. The broad peaks arising from Pt could be explained by the well-dispersed nature of the platinum particles, as demonstrated by the small particle size estimated using the Scherrer formula. A certain enhancement of the Pt-related reflections was evident after the reductive treatment, which suggested a slight particle size increase even at a relatively low temperature.

The EDS analysis over large sample areas resulted in composition values close to the nominal data for both the as prepared and reduced electrocatalysts. The Ti:Sn atomic ratio was approx. 4–5:1 (close to the nominal 4:1). The oxide:carbon weight ratio of 80–90:10–20 indicated that slightly more oxide was present in the analyzed area than the nominal value, while the Pt content was approx. 20–21 wt.%, which was in excellent agreement with the expectations. No composition change was detected in the reduced sample.

**Figure 2:**
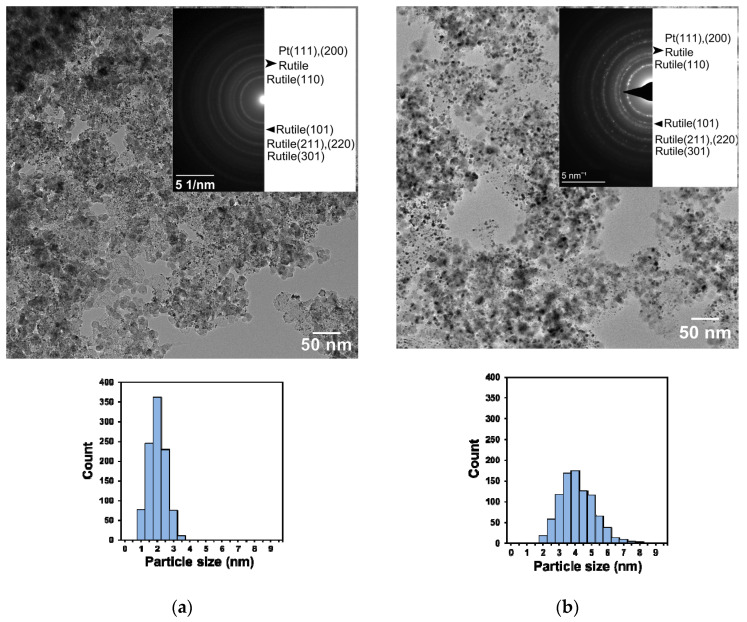
Low magnification TEM micrographs, electron diffraction patterns and Pt particle size distributions of (**a**) the as prepared Pt/Ti_0.8_Sn_0.2_O_2_-C electrocatalyst and (**b**) the Pt/Ti_0.8_Sn_0.2_O_2_-C electrocatalyst reduced at 200 °C.

An overview of low magnification TEM micrographs of the samples, as shown in Figure 2, evidenced the generally homogeneous appearance of the electrocatalysts. The small particles with dark contrast were identified as Pt while the oxide component had a much weaker contrast.

The electron diffraction patterns of both the as prepared and reduced catalysts were dominated by rings in the rutile phase. A broad band of overlapping rings arising from the lattice planes with d-spacings between 0.233–0.197 nm contained the most intense Pt-related features (the (111) and (200) rings at d-spacing around 0.227 nm and 0.196 nm, respectively). Face-centered cubic Sn–Pt alloys can also contribute to this region (e.g., Pt_3_Sn(111) at 0.231 nm [58]), along with weaker rutile reflections (such as (200), (111) or (210)). The diffraction patterns, therefore, confirmed the coexistence of rutile and Pt/alloy crystallites in both the as prepared and reduced systems.

The Pt particles were homogeneously dispersed in the as prepared catalyst and their size distribution was narrow with an average size of 2.3 ± 0.5 nm. The reduction at 200 °C initiated the enlargement of certain particles, leading to a broader size distribution and a shift of the average particle size to 4.4 ± 1.1 nm. Note that the evaluation of the XRD patterns essentially suggested the same crystallite sizes for Pt (Table 1).

**Figure 3:**
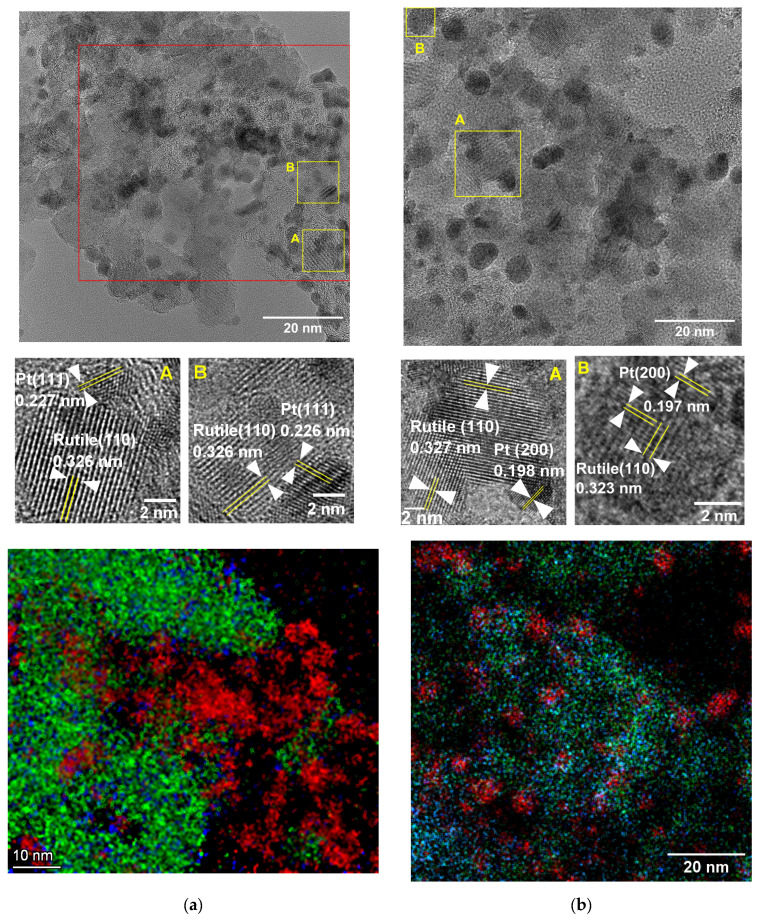
TEM micrographs and elemental maps for (**a**) the as prepared Pt/Ti_0.8_Sn_0.2_O_2_-C electrocatalyst and (**b**) the Pt/Ti_0.8_Sn_0.2_O_2_-C electrocatalyst reduced at 200 °C. The locations of the details A and B are indicated by yellow rectangles in the micrographs. The STEM/EDS elemental maps correspond to the area of the red rectangle in (**a**) and to the entire image area in (**b**). Color code: red: Pt, green: Ti, blue: Sn.

The microstructure of the as prepared and reduced catalysts, along with elemental maps of the depicted regions, is compared at higher magnification, as shown in Figure 3.

As far as the as prepared Pt/Ti_0.8_Sn_0.2_O_2_-C catalyst (Figure 3a) is concerned, the analysis of the lattice spacings and the comparison with the elemental maps confirmed that the roundish small dark objects were indeed Pt particles that homogeneously covered the catalyst surface. The 10–20 nm well-defined grey objects were rutile crystallites, which were faceted and relatively homogeneously covered the carbonaceous backbone. The d-spacings measured on these particles were very close to that of pure rutile (see details A and B), although the difference between the parameters of pure and slightly Sn-doped rutile was in the range of the uncertainties in the TEM measurements. As details A and B revealed, the Pt particles were very often closely associated with the oxide grains. The Ti and Sn content was concentrated into oxide particles (the maps of Ti, Sn and O (not shown) were almost congruent). The Ti:Sn ratio measured using STEM-EDS on the oxide particles (4–5:1) corresponded relatively well to the nominal value and the average value obtained over larger sample areas.

In fact, it was repeatedly observed in the literature of sol–gel synthesized Sn-doped TiO_2_ materials that the Sn introduction interferes with the rutile particle size growth during high-temperature treatments [59,60,61]. This effect was generally attributed to the formation of a segregated tin-oxide layer on the surface of the rutile crystallites. Therefore, the presence of a Sn-rich surface oxide would not be surprising in the systems investigated herein.

As already identified by the XRD investigations and particle size analysis, the most obvious consequence of the reductive treatment was a certain growth of the particles with dark contrast (Figure 3b). These particles retained the Pt-like face-centered cubic structure with lattice parameter changes that did not exceed the experimental uncertainties, although some Sn–Pt alloying could not be excluded. The rutile crystallites remained observable and retained their 10–20 nm size. However, they exhibited a rounder shape and their contour was more diffuse. As detail A demonstrated, the smaller metal particles were almost always closely associated with the oxide crystals. However, overlapping metal and oxide lattices were frequently characteristic for the larger Pt/alloy particles (detail B). The STEM-EDS elemental map indicated that while the distribution of Ti and Sn remained congruent, they appeared more homogeneously dispersed over the carbonaceous backbone, potentially suggesting a spreading of the oxide material during the reductive treatment.

It should be noted that Pt particles are usually more stable against particle size changes during low temperature annealing than observed herein. Early electrochemical investigations already revealed [62] and later TEM studies confirmed [63,64] that Pt nanoparticles deposited on different types of high surface area carbon supports are relatively stable below 300 °C annealing/reduction temperatures and noticeable particle size growth is induced only by temperatures exceeding 600 °C. In Pt/TiO_2_ systems, scant Pt particle growth was experienced after reductive pre-treatment up to 400 °C [65], under n-hexane reforming conditions up to 500 °C [66] or even during high-temperature vacuum annealing [67], demonstrating the nanoparticle stabilizing property of the support. At the same time, the introduction of tin can readily induce growth in Pt particles, and a significant increase in the particle size during the modification of platinum with tin was observed, leading to the formation of a Pt–Sn alloy [14]. Accordingly, we believe the interaction between Sn and Pt is responsible for the metal particle size growth experienced during the present low temperature reduction experiment.

### 3.2. Surface Chemistry of the as Prepared and Reduced Pt/Ti_0.8_Sn_0.2_O_2_-C Catalysts

#### 3.2.1. Surface Composition and Chemical States of the as Prepared and Reduced Catalysts

The XPS investigations were performed to explore the surface composition of the electrocatalysts and determine the bonding environment of the surface species. The analysis of these data was essential to obtain insight into the nature of the interaction between the oxide component of the support and the Pt particles. The Pt 4f spectra were evaluated as described in our other works [68]. The processing of the Sn 3d spectra involved correction for the satellite structure arising from the Sn 3d_3/2_ electrons excited by the MgKα_3,4_ radiation (the resulting spectral features overlap with the low binding energy region of the Sn 3d_5/2_ peak), followed by modeling the spectra with a 3d_5/2_-3d_3/2_ spin–orbit doublet with a symmetric peak shape for describing the ionic (Sn^4+^) contributions and with another doublet at lower binding energies for modeling the more reduced/metallic tin signals (if present).

In agreement with the results of the bulk-sensitive analytical methods (and the nominal composition), the Pt content of the catalysts measured using XPS was approx. 20–22 wt.% and the oxide:carbon weight ratio was close to 80:20. The actual composition data can be seen in Table S1 (Supplementary Materials). As the quantitative evaluation of the XPS data was based on the assumption of the homogeneous distribution of the components, this agreement confirmed the homogeneity of the samples on the characteristic length scale of XPS (~10 nm). This homogeneity was indeed observed on the electron micrographs (Figure 3). At the same time, XPS revealed Ti:Sn ratios at approx. 2:1 on the catalyst surface, instead of the 4–5:1 value characteristic for the bulk. This overrepresentation of Sn on the surface could be explained by the formation of a tin-rich overlayer covering the rutile crystals. Note that the XRD data, indicating a lower tin incorporation level than the nominal value (Table 1) as well as the disruption of the growth of the rutile crystallites observed by TEM, resulting in relatively small oxide particles (Figure 3) both pointed in the same direction. Importantly, neither XRD, electron diffraction patterns nor high-resolution TEM images contained direct indications regarding the tin-rich phase. Thus, we believe the overlayer was disordered and invisible for the diffraction-based techniques. The reductive pre-treatment caused no significant changes in the composition values (Table S1).

As expected for these systems, the carbon content of both catalysts was predominantly graphite-like with the most intense C 1s peak at 284.4 eV binding energy [69], accompanied by small signals from oxygen-bound carbon species. The O 1s spectra were always dominated by contributions due to metal oxides (Ti- and Sn-oxides) at approx. 530.3–530.5 eV binding energy [69], while a weaker high binding energy peak at approx. 531.5–532 eV indicated the presence of hydroxyl groups on the surface. Practically no difference was observed between the C 1s and O 1s spectra of the as prepared and reduced catalysts.

Table 2 summarizes the binding energies of the Pt 4f_7/2_, Sn 3d_5/2_ and Ti 2p_3/2_ core levels measured for the catalysts along with their assignments in the initial state (i.e., air-exposed without any in situ treatment in the electron spectrometer).

**Figure 4:**
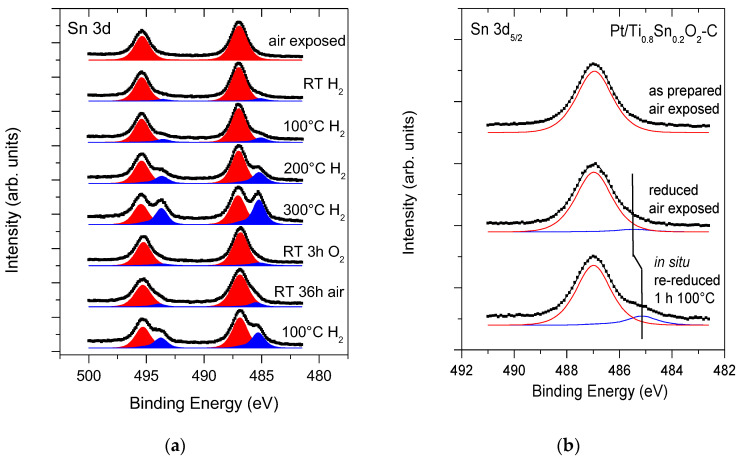
(**a**) Sn 3d spectra of the as prepared Pt/Ti_0.8_Sn_0.2_O_2_-C electrocatalyst collected during in situ treatments in the electron spectrometer. Hydrogen exposure was completed in 100 mbar H_2_ for 1 h at the indicated temperatures and oxygen exposure was conducted at 300 mbar at room temperature, while air exposure was performed at room temperature and ambient pressure. (**b**) The comparison of the Sn 3d_5/2_ peaks of the as prepared electrocatalyst in its initial (air-exposed) state, the 200 °C reduced electrocatalyst in its initial (air-exposed) state and the 200 °C reduced electrocatalyst after re-reduction in the electron spectrometer at 100 °C. (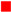): Sn^4+^ and (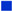): metallic or quasi-metallic Sn contribution.

The Pt content was predominantly metallic for both catalysts, although small ionic contributions were always observed, which were attributed to a certain degree of oxidation upon air exposure. The Pt 4f spectra of the as prepared and reduced catalysts are presented in Figure S1 in the Supplementary Materials. In all the cases, Ti was completely oxidized. The Sn 3d spectra were dominated by a 3d_5/2_-3d_3/2_ spin–orbit doublet feature with peaks at approx. 487.0 and 495.5 eV (Figure 4a and Figure 4b, uppermost trace). The doublet was assigned to fully oxidized Sn (Sn^4+^ ions) in a SnO_2_-like environment [69]. This assignment was confirmed by the observation of the Sn M_4_N_45_N_45_ Auger peak, which appeared at approx. 431.7–432.0 eV kinetic energy, corresponding to the Auger parameter values (sum of the Sn 3d_5/2_ binding energy and the Sn M_4_N_45_N_45_ kinetic energy) around 919 eV, in excellent agreement with the literature data for Sn^4+^ [69]. In addition to the dominant fully oxidized component, a rather weak reduced tin contribution was recognized in the spectrum of the air-exposed reduced electrocatalyst with a Sn 3d_5/2_ binding energy slightly above 485 eV (Figure 4b middle trace). Its assignment requires further considerations. While the reference data for Pt, TiO_2_, Sn or SnO_2_ are readily available in data bases [69], information on closely coupled Sn–Pt systems can be obtained from model experiments [71,72]. Based on these investigations, the Sn 3d_5/2_ binding energies of pure metallic tin and metallic Sn–Pt alloys seemed to be essentially identical. Additionally, a special tin state was identified that exclusively appears on oxidized alloy surfaces. This state is characterized by an intermediate binding energy between those of the metallic and ionic tin forms, namely, around 485.5 eV and is interpreted as tin species alloyed with surface Pt atoms but still partly O-bound [71,72,73]. In fact, in air-exposed Pt-Sn alloy nanoparticles, the lowest binding energy tin species is frequently observed around 485.4–485.6 eV. Therefore, it is usually identified as the Sn^0^ state, although it is sometimes noted that its binding energy is unusually high [11,19,74,75]. Considering this information, the weak reduced tin component observed in the spectrum of the reduced electrocatalyst was identified as this quasi-metallic tin state.

The dominance of the Sn^4+^ species at the surface of the electrocatalysts indicated that the (presumably) disordered tin-rich layer evidenced by the quantitative XPS measurements was completely oxidized.

#### 3.2.2. In Situ Reductive Treatment of the Electrocatalyst Samples

In order to obtain a more detailed insight into the interaction of Pt with the Sn-rich surface of the support, a series of in situ reduction treatments of the as prepared Pt/Ti_0.8_Sn_0.2_O_2_-C electrocatalyst was performed in the preparation chamber of the electron spectrometer at room and elevated temperatures. The reduction series was followed by oxygen and air exposure to assess the re-oxidation tendency of the materials, while the final step was a repeated reduction. The experiment was designed to model the reduction treatment (followed by the inevitable air exposure) for the preparation of the reduced Pt/Ti_0.8_Sn_0.2_O_2_-C electrocatalyst.

The Pt 4f spectra measured during this experiment revealed a complete reduction of Pt even after a slight hydrogen exposure. Otherwise, only marginal line shape changes were observed during the treatment series (Figure S1, Supplementary Materials).

The Sn 3d spectra collected during the in situ treatment series of the as prepared catalyst are shown in Figure 4a. In Figure 4b, the slight difference between the Sn 3d_5/2_ line shapes for the as prepared and reduced catalysts is emphasized, along with the spectral response of the latter system to a re-reduction treatment.

As already discussed, the symmetric and narrow Sn 3d peak shapes measured on the as prepared Pt/Ti_0.8_Sn_0.2_O_2_-C electrocatalyst in its initial air-exposed state, along with the Sn 3d_5/2_ binding energy around 487.0 eV, evidenced that Sn was completely oxidized (Figure 4a). Nevertheless, even after room temperature hydrogen exposure, a slight asymmetry emerged at the low binding energy part of the Sn 3d envelope, indicating the formation of a reduced component. The curve fitting revealed a Sn 3d_5/2_ binding energy of 484.8–485.0 eV for this new state, which accounted for 5% of the total Sn content. The binding energy of the reduced component was characteristic for metallic tin (Table 2 [69]). The elevation of the reduction temperature resulted in marginal shifts of the reduced Sn contribution, along with its continuous increase (10% at 100 °C, 20% at 200 °C and 40% at 300 °C). However, the described reduction of Sn was almost completely reversible. A decrease in the reduced tin signal to 10% of the total Sn intensity occurred after a few hours of room temperature O_2_ exposure, while the metallic Sn 3d_5/2_ peak remained slightly above the 485.0 eV binding energy. A longer (36 h) air exposure did not result in a further decrease in the reduced tin content. However, a shift in the 3d_5/2_ peak of the reduced component was observed at 485.5–485.6 eV, which indicated the formation of quasi-metallic tin species. Finally, a repeated reduction at 100 °C almost completely restored the situation observed before the oxidative treatments by increasing the reduced Sn content to 30%, accompanied by a shift in the metallic Sn 3d_5/2_ peak to 485.0–485.2 eV.

As shown in Figure 4b, the Sn 3d spectra of the as prepared electrocatalyst and its counterpart ex situ reduced at 200 °C in a H_2_ flow in the initial (air-exposed) state were rather similar, although a small contribution around 485.4–485.5 eV accounting for approx. 5% of the total intensity revealed the presence of quasi-metallic tin in the latter system. After a re-reduction of the latter sample in the electron spectrometer at 100 °C, the reduced Sn contribution increased to approx. 10% of the total tin signal and its 3d_5/2_ binding energy shifted to 485.1 eV, indicating the formation of metallic tin species.

A comparison between Figure 4a and Figure 4b confirmed the correspondence between the re-reduction behavior of the as prepared catalyst in situ reduced in the electron spectrometer and the ex situ reduced catalyst. Therefore, from a surface chemistry point of view, the two systems can be regarded as identical and the in situ reduction series modeled the changes induced by the reductive pretreatment.

In previous investigations, it was established that easy reduction of Sn-oxides to the metallic state was feasible only in the atomic vicinity of Pt sites [76,77,78]. Therefore, the appearance of reduced tin species even after room temperature hydrogen exposure confirmed the close contact of Pt with the tin-containing oxide component of the composite support, as already established by the TEM investigations. At the same time, according to the literature data [71,72,73] as well as to our own observations [11,19,79] on systems containing tin species on Pt, the formation of the quasi-metallic tin state as the result of oxidation could be used as a spectroscopic fingerprint of Sn–Pt alloying. Since the quasi-metallic state was not observed in the initial state of the as prepared catalyst, we believe that the Pt particles contained scant or negligible amounts of surface tin adsorbates and the tin species reduced during the first hydrogen exposure could be mostly identified as moieties bound around the perimeter of the Pt particles. A reduction at elevated temperatures certainly caused the migration of tin to the surface of the Pt particles, as evidenced by the appearance of the quasi-metallic state after air exposure.

**Figure 5:**
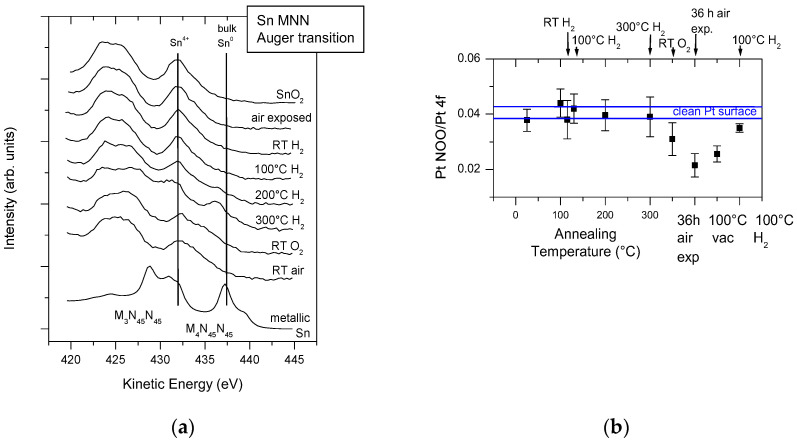
(**a**) Sn MNN Auger spectra for the as prepared Pt/Ti_0.8_Sn_0.2_O_2_-C electrocatalyst during in situ treatments in the electron spectrometer. For comparison, the reference spectra measured on SnO_2_ and the bulk metallic tin are also shown. (**b**) Pt NOO (64 eV)/Pt 4f (1180 eV) intensity ratio recorded at different stages of the in situ treatment series for the as prepared catalyst sample. Error bars represent the scatter in the ratio at a given treatment step, which was dominated by fluctuations in the measurements of the Pt NOO intensity.

Very similar conclusions were drawn from studying the development of the Sn MNN Auger peaks as well as the Sn 3d_5/2_–Sn M_4_N_45_N_45_ Auger parameter during the treatment series in the electron spectrometer. The Sn Auger spectra for the as prepared Pt/Ti_0.8_Sn_0.2_O_2_-C electrocatalyst are shown in Figure 5a. In the initial air-exposed state, the Sn MNN Auger spectrum was essentially identical to that of SnO_2_, with the Sn M_4_N_45_N_45_ peak at a kinetic energy of 432.0 eV (Auger parameter of 919.0 eV), which confirmed the fully oxidized nature of tin in the as prepared catalyst. While the completely oxidized signal at 432.0 eV kinetic energy was persistent during the reduction series (arising from ionic tin either incorporated into the rutile particles or at least located away from the Pt particles), a weak but clear component appeared in the kinetic energy range characteristic for metallic tin after the 100 °C step. After the reductive treatment at 200 °C and especially 300 °C, a well-defined peak emerged at a kinetic energy of 436.3 eV, which was considerably lower than that measured in the case of pure metallic tin (around 437.0 eV), resulting in an Auger parameter of 921.4–921.5 eV that was clearly below the characteristic value for metallic tin (approx. 922 eV). Since the kinetic energy of this peak and the resultant Auger parameter coincided with the values measured in the reduced Sn–Pt alloy catalysts [79], it was safe to conclude that during the 200–300 °C reduction, the transfer of tin to the Pt particles and subsequent tin–platinum alloying occurred in the composite-supported electrocatalyst. It is interesting to note that, due to the alloying, the state of tin after O_2_ or air exposure was different from the initial state of the catalyst. Even if the Sn 3d spectra suggested almost complete re-oxidation (apart from the small quasi-metallic fraction), the increased intensity of the Sn M_4_N_45_N_45_ Auger peak in the kinetic energy region above 432 eV evidenced the presence of a range of partially oxidized tin species. These were presumably protected from complete oxidation by their Pt-bound nature. The situation was analogous to the case of Sn–Pt alloy nanoparticles, where a broad Sn M_4_N_45_N_45_ Auger band at approx. 433.1 eV kinetic energy emerged after oxidation due to the formation of incompletely oxidized tin species on the surface of the alloy particles [19].

A particular feature of the weakly oxidized Sn–Pt surface alloys (which are mainly in the quasi-metallic state) is that they are readily reduced upon hydrogen exposure. The disordered monolayers are particularly reactive, resulting in a completely reduced tin state even at room temperature [73]. Reduction of thicker disordered tin-oxide layers may require elevated temperatures. It seems that reduction of these tin species occurs by the spillover of hydrogen from nearby Pt sites where hydrogen is activated.

In this sense, the response of the electrocatalysts for the surrounding atmosphere is reversible. Under oxidative conditions, a range of partially oxidized tin species is developed in the atomic vicinity of the Pt sites, which, under reductive conditions, are readily transformed into a metallic Sn–Pt alloy state. Sn species not associated to Pt were not found to be influenced by this reversible redox transition. Instead, they retain their fully oxidized nature. However, the transformation leading to Sn–Pt alloying—induced by the reductive treatment—is irreversible as the initial state of the catalysts could not be restored using mild oxidative treatments.

In fact, a known peculiarity of the Sn–Pt alloy systems is their dynamic response to the surrounding ambient. Earlier works [80,81] involving our own results [79] demonstrated that while Sn–Pt alloy nanosystems are metallic under reductive conditions, the segregation of tin to the surface accompanied by its oxidation occurs in oxidative environments.

In addition to the spectroscopic results discussed so far, a semiquantitative analysis also confirmed the reversible formation of a tin-oxide layer on the alloyed Pt particles during the in situ reduction/re-oxidation of the as prepared Pt/Ti_0.8_Sn_0.2_O_2_-C electrocatalyst. In this experiment, the variation of the intensity ratio of the low kinetic energy Pt NOO Auger transition and the high kinetic energy Pt 4f photoelectron peak was followed during the in situ treatment sequence. As described in Section 2.5, the intensity ratio is sensitive for the buried/exposed state of the Pt-containing particles. The observed variation of the intensity ratio is shown in Figure 5b. In the initial air-exposed state, a ratio close to that of a clean Pt surface was observed. It hardly changed during the reduction steps, indicating that Pt was a consistent surface species. However, the O_2_ exposure and especially the air exposure at the end of the reduction series resulted in significant decrease in the ratio, which was interpreted as a sign of burying Pt by a thin Sn-oxide overlayer. While 100 °C annealing in vacuum hardly influenced the ratio, its increase almost to the original value after a re-reduction at 100 °C indicated that the situation after the reduction steps could be restored.

To summarize this subsection, the surface spectroscopy investigations revealed considerable Sn–Pt alloying induced by a reductive treatment in the 100–300 °C range and a reversible encapsulation of the alloyed particles after air exposure by a Sn-oxide overlayer. However, the quantitative data suggested that the extent of Sn–Pt alloying was tuneable depending on the choice of the reduction temperature, which offers possibilities for the optimization of the functional properties of the catalysts.

Both the easy Sn–Pt alloying and the dynamic redox response of the alloys can be explained by the thermodynamics of the system. As the enthalpy of mixing Sn and Pt is strongly negative in the entire composition range [82], there is a significant driving force for alloying. Thus, fast mixing can be expected if metallic tin species (formed by a spillover of H activated on Pt) are in the atomic vicinity of Pt. On the other hand, the surface energy of SnO_2_, and even that of metallic Sn, is lower than that of pure Pt [83,84,85]. Therefore, there is a clear driving force for the segregation and oxidation of tin at the surface of the alloyed particles, in analogy to the classical strong metal-support interaction phenomenon. Since the activation energies for both the reduction and oxidation of tin on the alloyed surface are negligible [80], both processes are initiated spontaneously and immediately upon changing the atmosphere.

### 3.3. Electrocatalytic Properties of the As Prepared and Reduced Pt/Ti_0.8_Sn_0.2_O_2_-C Catalysts

According to the surface analytical results obtained during the in situ reduction series, the treatment at 200 °C resulted in less severe surface chemical changes compared to the treatment performed at 300 °C. Therefore, a hydrogen treatment temperature of 200 °C was selected for the preparation of the reduced electrocatalyst sample, as mentioned previously (see Section 2.3). The electrochemical performances of the as prepared and 200 °C reduced Pt/Ti_0.8_Sn_0.2_O_2_-C catalysts were compared using a three-electrode system. The performed tests involved cyclic voltammetry (CV), CO_ads_ stripping voltammetry and a stability assessment that used continuous cycling between 0.05–1.00 V potential limits.

**Figure 6:**
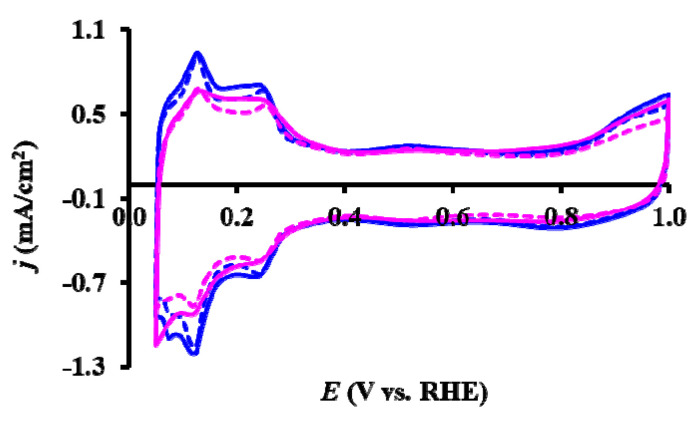
Cyclic voltammograms of the as prepared and reduced Pt/Ti_0.8_Sn_0.2_O_2_-C composite-supported electrocatalysts. The voltammograms were recorded in 0.5 M H_2_SO_4_ before (solid curves) and after 500 cycles (dashed curves) of the stability test. Polarization rate: 100 mV/s. As prepared catalyst: (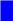), reduced catalyst: (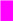).

Figure 6 summarizes the cyclic voltammograms of the as prepared and 200 °C hydrogen reduced electrocatalysts obtained before and after the short 500-cycle stability test runs. Certain electrochemical features derived from the voltammograms are listed in Table 3. Briefly, the voltammograms were dominated by the desorption/adsorption peaks of underpotentially deposited hydrogen in the 50–350 mV potential range and the oxidation/reduction of Pt above 800 mV. Additionally, in the double layer region (between 300–700 mV), weakly expressed peaks of the quinone–hydroquinone redox couple at 550 mV were also detected. The quinone-type-oxygen group usually appears on the surface of carbonaceous materials oxidized with nitric acid [86,87], involving e.g., MWCNTs-supported Pt/SnO_x_ composite catalysts [88]. It should be noted that we observed a similar shape of CVs on the Pt electrocatalysts supported on TiO_2_-C hybrid materials (C: Black Pearls 2000 and graphite oxide (GO) derived carbon) that were prepared using a similar procedure [89].

In addition to the quinone-type-oxygen groups, the redox peaks in the double layer region can also be associated with tin species. The redox peaks reported previously in model Sn–Pt systems [90,91] observed at 400–600 mV were usually attributed to the Sn(II)/Sn(IV) redox couple for tin-oxide/hydroxide species immobilized onto Pt. It should be noted that these Sn(II)/Sn(IV) redox peaks were much more pronounced and depended on the surface concentration of tin. This is not our case. Our preliminary experiments indicate the independence of the magnitude of these peaks from the surface concentration of tin [48]. It should be noted that an assumption was also made about the possibility of attributing these surface redox pairs to the adsorption of sulfate anions [90,91]. However, based on the results of model Sn–Pt catalytic systems measured in various electrolytes, this hypothesis was not confirmed.

The voltammograms presented in Figure 6 are similar to those reported for other Ti–Sn mixed oxide-containing Pt/Ti_(1−x)_Sn_x_O_2_ electrocatalytic systems [25,92]. In particular, the electrochemically active surface area (ECSA) deduced from the underpotentially deposited hydrogen adsorption/desorption peaks (Table 3) is comparable to those mentioned in the literature (40 to 60 m^2^ g^−1^) [92].

In addition, the CVs of the as prepared and H_2_ treated samples had similar features, although the reduced sample showed a slight decrease in the hydrogen adsorption/desorption peaks compared to those obtained on the as prepared system. As a result, the initial ECSA value of the as prepared catalyst after reduction decreased from 53.2 to 39.7 m^2^/g_Pt_ (Table 3). The ECSA loss in this system due to reduction can be attributed to the combined effect of the broadening of the Pt size distribution (formation of larger particles during the reduction, see the TEM results) and the encapsulation of the alloyed particles by segregated Sn-oxides (as suggested by the electron spectroscopy results).

**Figure 7:**
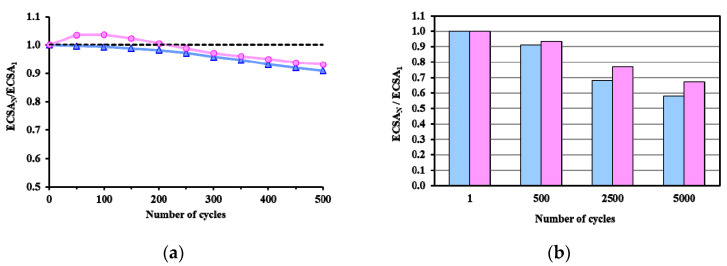
ECSA change during 500 CV cycles (**a**) and 5000 CV cycles (**b**) for the as prepared (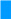) and reduced (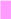) Pt/Ti_0.8_Sn_0.2_O_2_-C electrocatalysts. ECSA_N_/ECSA_1_: comparison of the ECSA measured after N cycles normalized to the ECSA measured in the first cycle of both the as prepared and H_2_-reduced catalyst as a function of the number of cycles (N).

The performance of both the as prepared and H_2_-reduced electrocatalyst was studied using short 500-cycle and long-term 5000-cycle electrochemical stability tests (Table 3 and Figure 7). The values of the electrochemically active Pt surface area loss (ΔECSA) as a function of the number of cycles of the stability test are presented in Table 3. In the case of the as prepared catalyst, a continuous ECSA decrease approaching 10% by the end of the 500-cycle test (Figure 7a) and more than 40% after the long-term test (Figure 7b) was recorded. On the other hand, an initial ECSA increase was observed for the reduced sample at the beginning of the short test (Figure 7a). It can be interpreted as the cleaning of the catalyst surface from residual impurities or oxides that cause the blocking effect [45,93,94].

It should be noted that we previously observed similar behavior on the alloy-type Sn–Pt/C electrocatalysts prepared using controlled surface reactions [19]. In analogy with the results of the surface chemistry investigations described above, it was suggested that after the contact of the alloy-type Sn–Pt/C samples with air, a site-blocking effect arose due to the segregation of tin, probably in the form of a thin layer of SnO_x_ over the Pt–Sn alloy phase (and/or Pt), which was the reason for a pronounced decrease in the H_2_ adsorption/desorption. It is known that SnO_2_ suffers from poor stability at high potentials since SnO_2_ can dissolve under acidic conditions [95]. It has been shown [11,19] that with an increase in the number of cycles of cyclic polarization, there is a slight increase in the value of the ECSA, which is associated with the cleaning of the electrode surface from SnO_x_ species.

The initial ECSA increase in the reduced catalyst turned into a decrease after approx. 100 cycles. Nevertheless, the decrease remained moderate and the relative ECSA loss was always smaller than that of the as prepared sample, indicating the stability enhancement effect of the reductive pre-treatment. However, the calculation of the ECSA from the hydrogen adsorption/desorption region included some uncertainties. It is known that capacitive currents, which originate from double layer charging, have to be subtracted before H_UPD_ charges can be calculated. Different methods of background current correction can be used, such as (i) a constant capacitive current in the H_UPD_ potential range (a straight line correction) or (ii) the CO stripping curve baseline correction (which yields to considerably higher H_UPD_ charges) [96,97]. Both methods exhibited inaccuracies, but the difference between the as prepared and reduced catalysts at the end of the test was large enough to demonstrate the beneficial effect of the reductive treatment.

**Figure 8:**
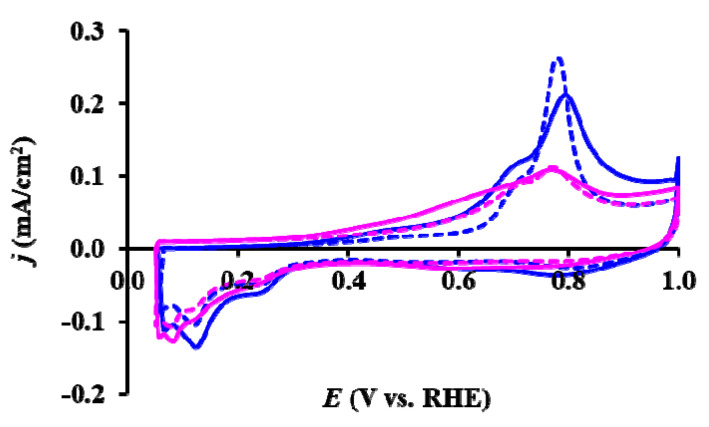
CO_ads_ stripping voltammograms of the as prepared and reduced Pt/Ti_0.8_Sn_0.2_O_2_-C composite-supported electrocatalysts. The voltammograms were recorded in 0.5 M H_2_SO_4_ before (solid curves) and after 500 cycles (dashed curves) of the stability test. Sweep rate: 10 mV/s. As prepared catalyst: (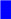), reduced catalyst: (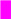).

For the investigation of the CO tolerance of the catalysts and to obtain electrochemical information about the surface chemistry of the system, the oxidation of CO adsorbed (CO_ads_) on the catalyst surface from the CO-saturated electrolyte was studied. The CO_ads_ stripping voltammograms for the as prepared and reduced catalysts obtained before and after 500 cycles of the stability test are shown in Figure 8.

As shown in Figure 8, the CO_ads_ oxidation on these catalysts resulted in a broad feature containing a shoulder and a main peak from ca. 200 to 850 mV. As presented in Table 3, for both samples, the onset potential of CO electrooxidation (E_CO,onset_) was approx. 200 mV. A slight difference was observed in the position of the maximum of the main CO oxidation peak (795 mV for the as prepared catalyst and 765 mV for the reduced catalyst), while the shoulder appeared at 695 mV in both cases.

According to the literature, the oxidation of CO on Sn-modified Pt/C catalysts follows a bifunctional mechanism in which CO is adsorbed onto Pt, while Sn sites nucleate OH_ads_ species at less positive potentials than Pt [98,99]. In this regard, it should be emphasized that CO electrooxidation on Pt-based electrodes also occurs in the presence of OH_ads_ species, which are formed on Pt/C in an acidic medium at E > 600 mV, but are available on the surface of Sn-containing electrodes at potentials less than 100 mV.

It is well documented for the Pt/SnO*_x_*/C catalysts that the CO oxidation peaks significantly broaden and split into at least two components. The peak at higher potential is usually attributed to the CO oxidation on Pt, while the lower potential shoulder (≤700 mV) is ascribed to the CO oxidation on Pt close to the interface with adjacent tin-oxide species [100]. In our previous studies [19], the CO_ads_ stripping peak at ~700 mV was attributed to CO electrooxidation on the Sn–Pt/C alloy-type electrocatalysts. In ref. [101], the CO_ads_ oxidation began at approx. 200 mV and exhibited only a single peak centered at ~640 mV on the commercial Pt_3_Sn alloy catalyst (20 wt.%, E-TEK). Further studies of platinum catalysts supported on tin oxide (Pt/SnO*_x_*) confirmed the CO_ads_ oxidation onset potentials below 400 mV, indicating an increase in the CO electrooxidation capability compared to state-of-the-art CO-tolerant PtRu/C catalysts [46]. Compared to these data, additional positive values for both the E_CO,onset_ (707 mV) and E_CO,max_ (839 mV) were obtained on a Ti_0_._9_Sn_0_._1_O_2_-C composite-supported Pt electrocatalyst by Li et al. [92]. Accordingly, the electrochemical behavior of both the as prepared and reduced Pt/Ti_0.8_Sn_0.2_O_2_-C composite-supported electrocatalysts reflected the known characteristics of the Pt/SnO*_x_*-containing systems, confirming the importance of direct Sn–Pt interactions in determining the catalytic properties.

Although the onset potential for CO_ads_ oxidation as well as the potentials of the features of the main CO oxidation envelope remained close in the reduced catalyst to those in the as prepared sample, an essential change in the shape of the CO_ads_ stripping voltammogram was evident. The suppression of the main peak as well as the relative enhancement of the shoulder region resulted in a voltammogram shape resembling those observed for the Sn–Pt alloy electrocatalysts [19]. This observation was in agreement with the conclusions drawn from the surface analytical investigations, which also suggested Sn–Pt alloying upon reduction.

As shown in Figure 8, after the 500-cycle stability test of the as prepared catalyst, the currents related to low-potential CO oxidation became less pronounced, and a certain shift (~10–20 mV) of the main peak was observed towards less positive potential values compared to that obtained on the fresh sample. It is possible that this shift was associated with some agglomeration of Pt nanoparticles [102]. The qualitatively similar but more moderate changes in the voltammogram of the reduced sample, along with unchanged peak positions, identified its enhanced stability. The changes induced by cycling (involving decreasing currents in the pre-peak region and a sharpening of the main peaks) may point to the dissolution/deactivation of certain tin species responsible for the CO tolerance, although the resistance of both the as prepared and reduced catalysts against CO poisoning was maintained during the 500-cycle stability test.

## 4. Conclusions

Pt electrocatalysts were prepared on a novel Ti_0.8_Sn_0.2_O_2_-C-type composite support in order to assess their performance as corrosion resistant replacements for Pt/C in PEMFC applications and to identify the effect of reductive pre-treatment on the catalytic behavior.

Structural investigations evidenced a good coverage of the carbon backbone by the oxide phase and TEM images confirmed a close connection between the Pt particles and the oxide crystallites. At the same time, the XRD data suggested a moderate tin incorporation into the mixed oxide phase and XPS indicated the presence of a tin oxide-rich overlayer. The XPS and electrochemical investigations both evidenced a strong coupling between the surface Sn species and Pt.

The microstructure of the catalyst allowed for the anchoring of the Pt nanoparticles at the oxide–carbon interface, leading to the formation of Pt–oxide–carbon triple junctions at a high density, which was a prerequisite for high dispersion, stability and activity.

Our assumption was that reductive pre-treatment could further enhance this coupling by initiating a solid state migration of support oxide-related species to the surface of the Pt particles in analogy with strong metal-support interaction. The electrochemical stability tests revealed a better long-term stability for the reduced system. At the same time, the XPS and electrochemical investigations identified that an important structural change, Sn–Pt alloying, occurred during the reductive treatment, which was the combined result of the intimate connection of Pt and Sn surface species and the thermodynamics of the Sn–Pt system. A consequence of the alloying was the reversible partial encapsulation of the metal particles by a tin-oxide overlayer under oxidative conditions.

The presence of quasi-metallic tin species proved to be an excellent descriptor for the alloying between Sn and Pt.

As the extent of alloying can be influenced by treatment parameters such as the reduction temperature, the reductive pre-treatment can be identified as a suitable method for modulating the metal-support interaction in these and other tin-oxide-containing Pt-based electrocatalysts.

## Figures and Tables

**Table 1:** Characterization of the Ti_0.8_Sn_0.2_O_2_-C composite support and the as prepared and reduced Pt/Ti_0.8_Sn_0.2_O_2_-C electrocatalysts using XRD.

Sample ID	Crystallite Sizes (nm) ^a^		Lattice Parameters, Å ^b^	Sn Substitution, % ^c^
	Rutile	Pt		
Ti_0.8_Sn_0.2_O_2_-C composite	12	n/a	*a* = 4.61, *c* = 2.98	~10
Pt/Ti_0.8_Sn_0.2_O_2_-C as prepared	10	3.3	*a* = 4.61, *c* = 2.98	~10
Pt/Ti_0.8_Sn_0.2_O_2_-C reduced	8–9	4.1	*a* = 4.62, *c* = 2.99	~10–20

^a^ Estimated by the Scherrer formula; ^b^ lattice parameters of the rutile phase (pure rutile TiO_2_: *a* = 4.593 Å, *c* = 2.959 Å), ^c^ relative amount of incorporated Sn determined by Vegard’s law [56].

**Table 2:** Core level binding energies for the Pt 4f_7/2_, Sn 3d_5/2_ and Ti 2p_3/2_ peaks (in the initial air-exposed state).

Core Level	As Prepared	Reduced	Assignment
	Binding Energy (eV) ^a^		
Pt 4f_7/2_	71.3 (93%)	71.3 (89%)	metallic Pt [69]
	72.6 (4%)	72.6 (9%)	Pt^2+^: PtO, Pt(OH)_2_ [70]
	75.1 (3%)	75.1 (2%)	Pt^4+^: PtO_2_ [70]
Sn 3d_5/2_	487.0 (100%)	485.4 (5%)	(quasi)metallic Sn
		486.9 (96%)	Sn^4+^: SnO_2_ [69]
Ti 2p_3/2_	459.1	459.0	Ti^4+^: TiO_2_ [69]

^a^ numbers in parentheses indicate the relative contribution of the given chemical state.

**Table 3:** Electrochemical performance of the as prepared and reduced Pt/Ti_0.8_Sn_0.2_O_2_-C catalysts.

Sample ID	E_CO,onset_, ^a^ mV	E_CO,max_, ^b^ mV	ECSA_1_, ^c^ m^2^/g_Pt_	ΔECSA_500_, % ^d^	ΔECSA_5000_, % ^d^
As Prepared Pt/Ti_0.8_Sn_0.2_O_2_-C	~200	795 (*sh*: 695)	53.2	9.1	41.9
		775 (*sh*: 695) *			
Reduced Pt/Ti_0.8_Sn_0.2_O_2_-C	~200	765 (*sh*: 695)	39.7	6.8	32.9
		765 (*sh*: 695) *			

^a^ The onset potential for the CO electrooxidation; ^b^ the position of the main CO stripping peak measured on fresh catalysts and after the 500-cycle stability test (indicated by *); ^c^ the ECSA_1_ value obtained on fresh catalysts; ^d^ ΔECSA_500_ and ΔECSA_5000_ were calculated from the charges originated from the hydrogen desorption in the first and N= 500th or 5000th cycles, according to the Equation ΔECSA_N_ = {1 − (ECSA_N_/ECSA_1_)} × 100%; *sh* = shoulder.

## Data Availability

The data presented in this study are available on request from the corresponding author.

## Supplementary Materials

The following supporting information can be downloaded at: https://www.mdpi.com/article/10.3390/nano13152245/s1, Figure S1: Pt 4f spectra of the as prepared and reduced electrocatalysts; Table S1: composition of the as prepared and reduced electrocatalysts determined using XPS.
