# Supplementary material for: Reductive Treatment of Pt Supported on Ti0.8Sn0.2O2-C Composite: A Route for Modulating the Sn–Pt Interactions
Source: Nanomaterials (Basel). 2023 Aug 3;13(15):2245. doi: 10.3390/nano13152245 (PMC10473237; doi:10.3390/nano13152245)
Supplement: Supplementary file 1 [file nanomaterials-13-02245-s001.zip › nanomaterials-2525110-supplementary-final.pdf]

Supplementary Materials

for

Reductive Treatment of Pt Supported on $Ti_{0.8}Sn_{0.2}O_2$-C Composite: A Route for Modulating the Sn–Pt Interactions

Cristina Silva [1,2], Khirdakhanim Salmanzade [1], Irina Borbáth [1], Erzsébet Dódony [3], Dániel Olasz [3], György Sáfrán [3], Andrei Kuncser [4], Erzsébet Pászti-Gere [5], András Tompos [1], Zoltán Pászti [1,*]

[1] Institute of Materials and Environmental Chemistry, Research Centre for Natural Sciences, Magyar Tudósok Körútja 2, H-1117 Budapest, Hungary
[2] Department of Physical Chemistry and Materials Science, Faculty of Chemical Technology and Biotechnology, Budapest University of Technology and Economics, Műegyetem rkp. 3., H-1111 Budapest, Hungary
[3] Institute for Technical Physics and Materials Science, Centre for Energy Research, Konkoly-Thege Miklós út 29-33, H-1121 Budapest, Hungary
[4] National Institute of Materials Physics, 405A Atomistilor Street, 077125 Magurele, Romania
[5] Department of Pharmacology and Toxicology, University of Veterinary Medicine, István utca 2, H-1078 Budapest, Hungary
* Correspondence: paszti.zoltan@ttk.hu; Tel.: +36-1-3826-412

The Supplementary Materials section contains the following information:

- **Figure S1**: Pt 4f spectra of the as prepared Pt/$Ti_{0.8}Sn_{0.2}O_2$-C catalyst collected during the *in situ* treatment series and comparison of the Pt 4f spectra of the as prepared and reduced catalysts;
- **Table S1**: Composition data for the as prepared Pt/$Ti_{0.8}Sn_{0.2}O_2$-C catalyst collected by XPS during the *in situ* treatment series, completed with ones obtained from the reduced Pt/$Ti_{0.8}Sn_{0.2}O_2$-C system.

In Figure S1 (A) Pt 4f spectra collected during the *in situ* treatment series of the as prepared $Pt/Ti_{0.8}Sn_{0.2}O_2$-C catalyst (Section 3.2.2) are shown. As additional information, a comparison of the Pt 4f spectra for the as prepared electrocatalyst in its initial (air exposed) state, the 200 °C reduced electrocatalyst in its initial (air exposed) state and the 200 °C reduced electrocatalyst after re-reduction in the electron spectrometer at 100 °C is presented in Figure S1 (B).

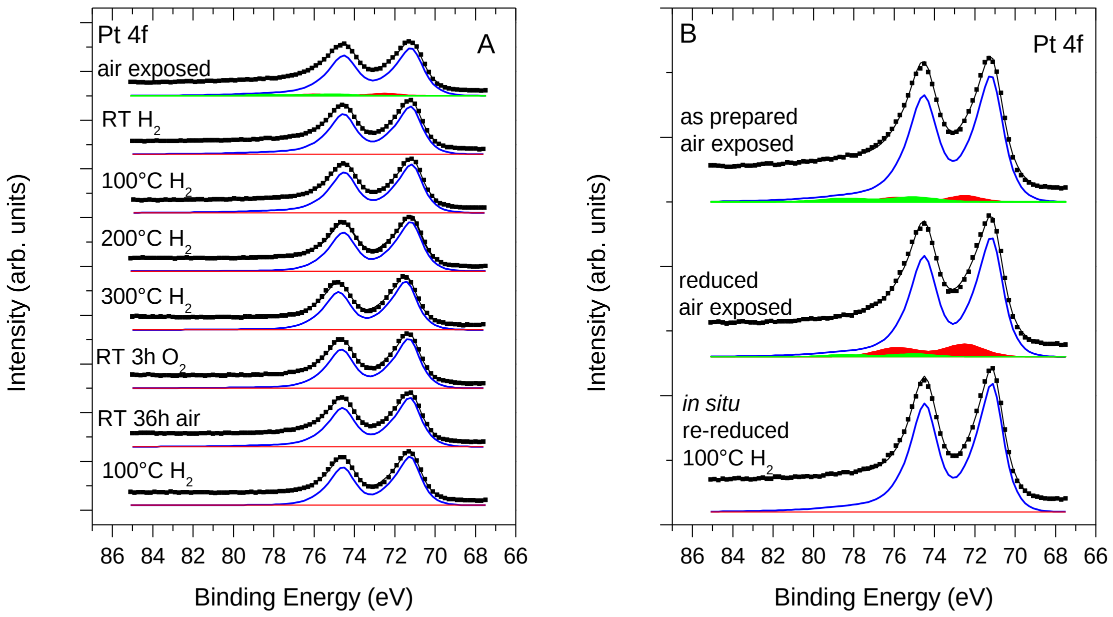

Figure S1. **(A)**: Pt 4f spectra of the as prepared $Pt/Ti_{0.8}Sn_{0.2}O_2$-C electrocatalyst collected during *in situ* treatments in the electron spectrometer. Hydrogen exposure was completed in 100 mbar H_2 for 1 h at the indicated temperatures, oxygen exposure was done at 300 mbar at room temperature, while air exposure was performed at room temperature and ambient pressure. **(B):** comparison of the Pt 4f peaks of the as prepared electrocatalyst in its initial (air exposed) state, the 200 °C reduced electrocatalyst in its initial (air exposed) state and the 200 °C reduced electrocatalyst after re-reduction in the electron spectrometer at 100 °C. Dots: data, black line: fit, (■): metallic Pt and (■): ionic Pt (Pt^{2+}), (■): ionic Pt (Pt^{4+})

Pt 4f spectra were modeled by an asymmetric spin-orbit doublet line shape which was measured on a reduced Pt/C reference catalyst with 40 wt% Pt content; this contribution represents the metallic Pt signal. In the initial, air exposed state of the catalysts small additional doublets were necessary for adequate fit, which were assigned to ionic Pt contributions (for details, see Table 2 of the manuscript).

As it is described in the text (Section 3.2.1), the Pt content of both the as prepared and the reduced $Pt/Ti_{0.8}Sn_{0.2}O_2$-C catalysts was predominantly metallic, accompanied by a small amount of ionic (Pt^{2+} and Pt^{4+}) species. Only a slight hydrogen exposure was enough to reduce the ionic Pt species. In this sense the initial, air exposed states of the as prepared and reduced catalysts were very similar. Minimal line shape changes and negligible shifts were observed during the *in situ* treatment series, confirming that Sn-Pt alloying is difficult to detect based on the Pt core level spectra.

Composition data for the as prepared $Pt/Ti_{0.8}Sn_{0.2}O_2$-C catalyst derived from XPS spectra collected during the *in situ* treatment series, completed with ones obtained from the reduced $Pt/Ti_{0.8}Sn_{0.2}O_2$-C system, are summarized in Table S1.

Table S1. Composition and atomic ratio data for the as prepared $Pt/Ti_{0.8}Sn_{0.2}O_2$-C catalyst measured during the *in situ* treatment series by XPS. Data for the reduced $Pt/Ti_{0.8}Sn_{0.2}O_2$-C catalyst in its initial state and after 100°C re-reduction in the electron spectrometer are also shown.

Catalyst, treatment	Composition (atomic %)					Atomic ratio	
	Pt	Ti	Sn	O	C	Ti:Sn	Pt:Sn
As prepared $Pt/Ti_{0.8}Sn_{0.2}O_2$-C Initial state	3.2	12.5	6.1	41.9	36.3	2.05:1	0.53:1
As prepared $Pt/Ti_{0.8}Sn_{0.2}O_2$-C Room temp. H_2	3.6	13.3	6.6	40.5	36.0	2.01:1	0.54:1
As prepared $Pt/Ti_{0.8}Sn_{0.2}O_2$-C 100°C H_2	3.6	13.3	6.6	39.4	37.1	2.01:1	0.54:1
As prepared $Pt/Ti_{0.8}Sn_{0.2}O_2$-C 200°C H_2	3.4	14.3	7.0	38.5	36.8	2.03:1	0.48:1
As prepared $Pt/Ti_{0.8}Sn_{0.2}O_2$-C 300°C H_2	3.3	15.7	5.7	38.5	36.8	2.75:1	0.57:1
As prepared $Pt/Ti_{0.8}Sn_{0.2}O_2$-C Room temp. O_2	2.8	14.8	5.7	40.1	36.6	2.56:1	0.49:1
As prepared $Pt/Ti_{0.8}Sn_{0.2}O_2$-C Room temp. air	2.6	14.4	5.4	42.0	35.6	2.66:1	0.48:1
As prepared $Pt/Ti_{0.8}Sn_{0.2}O_2$-C Re-red. 100°C H_2	2.9	15.2	5.8	40.3	35.8	2.65:1	0.51:1
Reduced $Pt/Ti_{0.8}Sn_{0.2}O_2$-C Initial state	3.5	12.5	5.5	38.1	40.4	2.27:1	0.64:1
Reduced $Pt/Ti_{0.8}Sn_{0.2}O_2$-C Re-red. 100°C H_2	3.4	11.6	5.2	37.6	42.2	2.23:1	0.65:1

As described in the main text, the compositions correspond quite well to the nominal composition. The slight composition difference between the as prepared and the reduced $Pt/Ti_{0.8}Sn_{0.2}O_2$-C catalysts (somewhat higher carbon content in the latter case and slightly different Ti:Sn ratio) can be ascribed to sample-to-sample variation. The data set obtained during the *in situ* treatment series of the as prepared catalyst (see Section 3.2.2) reveals variations within the range of the uncertainities of the XPS measurement; maybe a certain change of the Ti:Sn ratio at the end of the reduction series and the marginal decrease of the apparent Pt content after oxidative treatments can suggest some structural rearrangement. These data demonstrate that the intensity ratio of the low/high kinetic energy Pt features is a much better indicator of structural changes of the catalyst.
